# Supplementary material for: Mtfp1 ablation enhances mitochondrial respiration and protects against hepatic steatosis
Source: Nat Commun. 2023 Dec 20;14:8474. doi: 10.1038/s41467-023-44143-9 (PMC10733382; doi:10.1038/s41467-023-44143-9)
Supplement: Supplementary file 1 — Supplementary Information [file 41467_2023_44143_MOESM1_ESM.pdf]

# Supplementary information

# *Mtfp1* ablation enhances mitochondrial respiration and protects against hepatic steatosis

Cecilia PATITUCCI, Juan Diego HERNÁNDEZ-CAMACHO, Elodie VIMONT, Sonny YDE, Thomas COKELAER, Thibault CHAZE, Quentin GIAI GIANETTO, Mariette MATONDO, Anastasia GAZI, Ivan NEMAZANYY, David A. STROUD, Daniella H. HOCK, Erminia DONNARUMMA, Timothy WAI.

## Description of additional Supplementary files

**Supplementary information:**

Supplementary figures 1-9

Supplementary Dataset 1

Supplementary Dataset 2

Supplementary Dataset 3

Supplementary Dataset 4

Supplementary Dataset 5

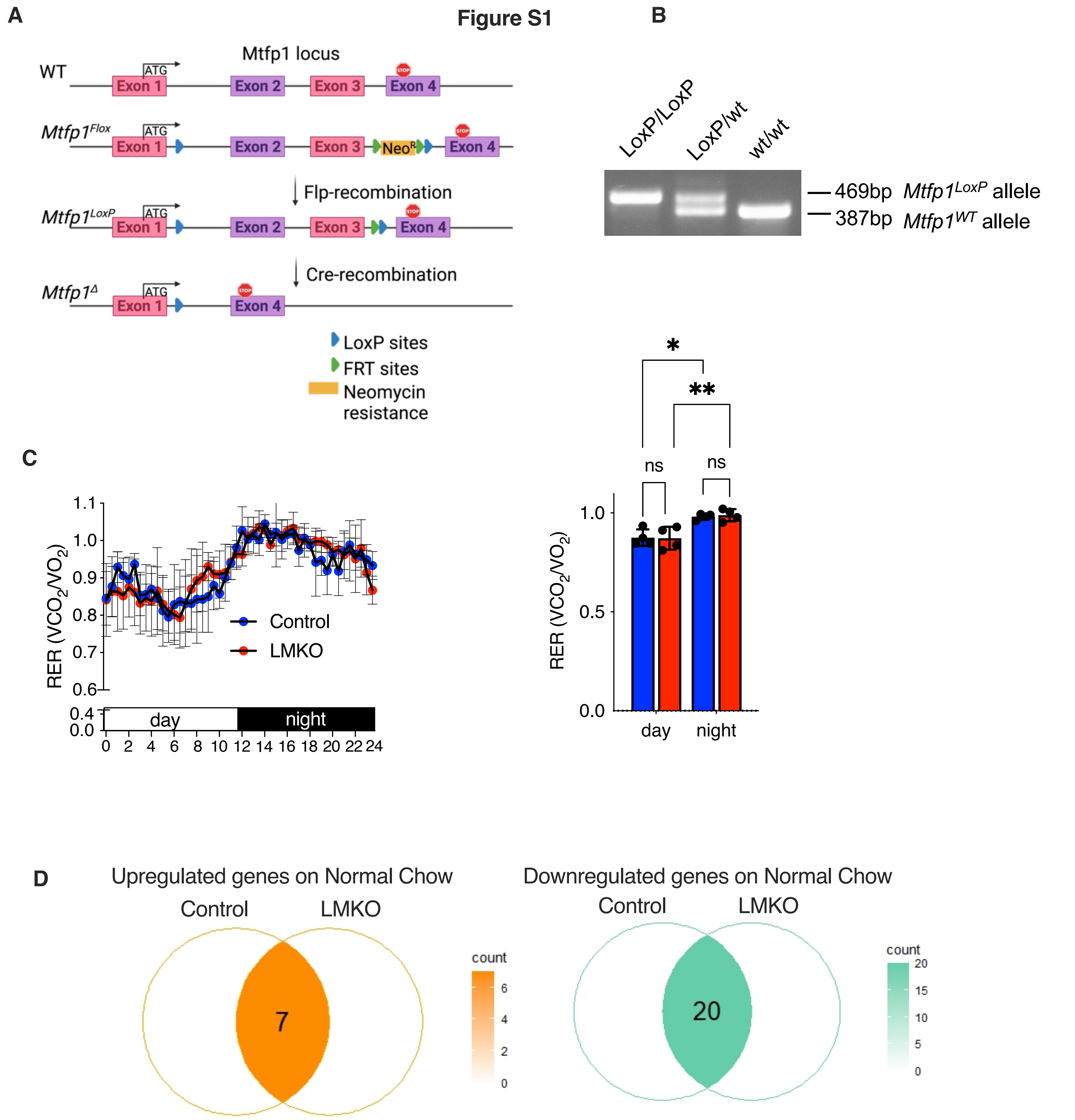


**Figure S1. Generation and characterization of *Mtfp1* liver-specific knockout mice**

**A)** Targeting strategy for conditional inactivation of mouse *Mtfp1* in LMKO mice. To allow detection of *Mtfp1* exons 2 and 3 were flanked in both cases by LoxP sites (blue arrowheads). Flox denotes NeoR cassette containing LoxP targeted locus. LoxP denotes NeoR cassette-deleted targeted locus. Δ denotes deletion induced by Cre-recombinase. FRT sites (green) initially flank NeoR cassette (yellow). Exons 2 and 3 were deleted by Cre-recombinase. Created with Biorender.com

**B)** Genotyping PCR for the distal LoxP site in the conditional *Mftp1* locus from genomic DNA. The wild type (WT; 387 bp) and the conditional (LoxP; 469 bp) alleles are shown for $Mtfp^{WT/WT}$, $Mtfp1^{LoxP/WT}$, and $Mtfp1^{LoxP/LoxP}$ mice.

**C)** Respiratory Exchange Ratio (RER) for control and LMKO male mice on fed a normal chow diet (NCD). RER analyses for 24 hours are shown. Mean values for day and night periods. n=4. Data are means ± SD. 2-tailed, unpaired Student's t test. *: $p<0.05$. **: $p<0.01$ ns=not significant.

**D)** Upregulated (orange) and downregulated (cyan) genes in LMKO mice on a normal chow diet corresponding to Supplemental Dataset 1.

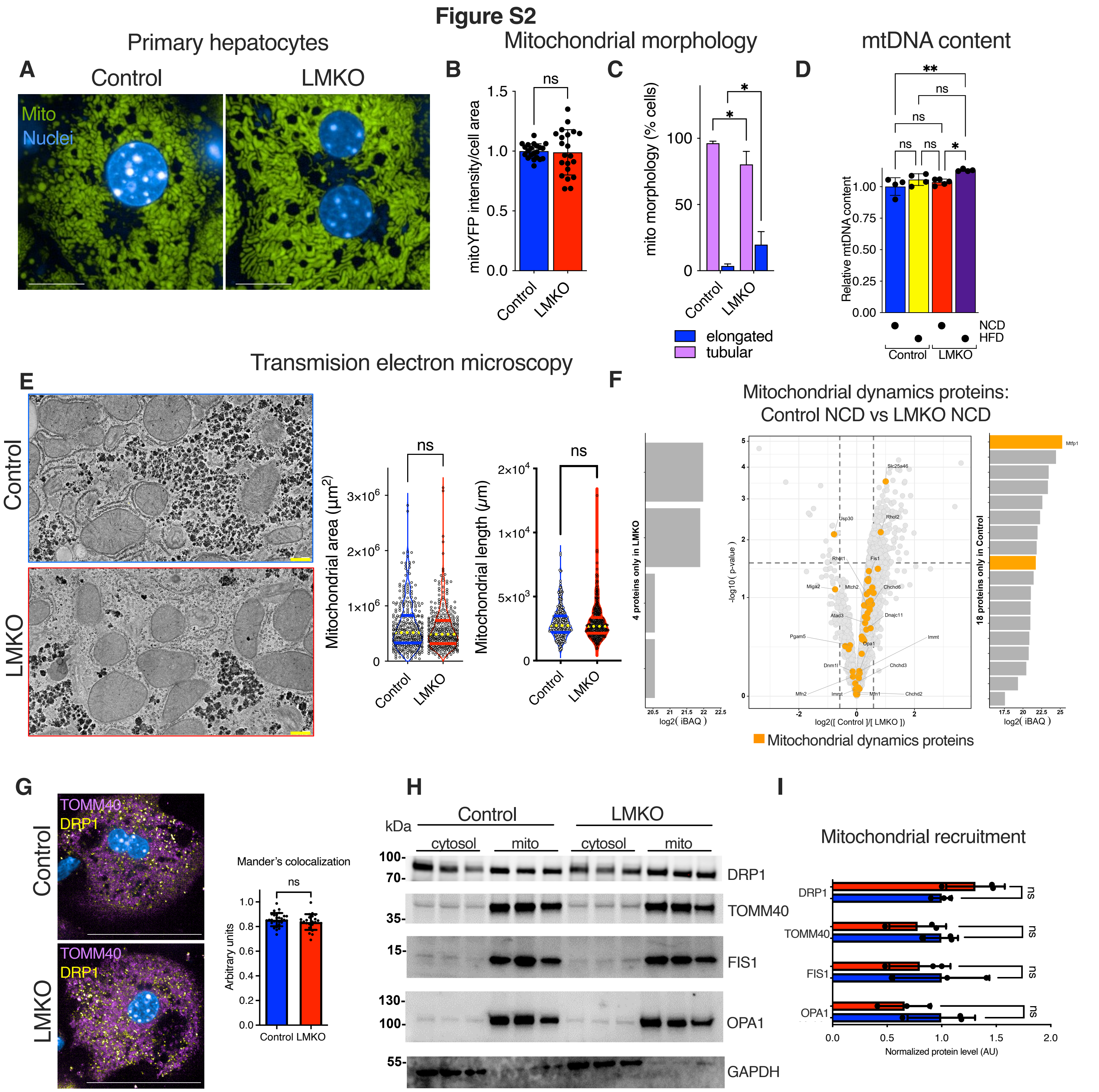


**Figure S2. Impact of *Mtfp1* deletion on mitochondrial mass and morphology**

**A)** Representative images of primary hepatocytes isolated from control and LMKO female mice expressing mitoYFP (green). Nuclei in blue. Scale bar=100$\mu$m.

**B)** Quantification of mitochondrial imaging in primary hepatocytes from control and LMKO expressing mitoYFP mice. Single cell mitoYFP intensity is normalized to hepatocyte area. 2-tailed, unpaired Student's t test, ns=not significant.

**C)** Quantification of mitochondrial morphology in primary hepatocytes from control and LMKO female mice performed by supervised machine learning. 2-tailed, unpaired Student's t test, *: p<0.05.

**D)** Mitochondrial DNA (mtDNA) content measured by qPCR relative to nuclear DNA in livers from control and LMKO male mice fed a normal chow (NCD) or high-fat (HFD) diets for 16 weeks. n=4. Data are means ± SD. 2-way ANOVA, *: p<0.05. **: p<0.01, ns=not significant.

**E)** Mitochondrial surface, area and length determined by transmission electron microscopy (TEM) of control and LMKO liver sections. Median (yellow dotted line) and Quartiles (solid colored line). Scale bar=500$\mu$m. Control n= 322. LMKO n= 341. Data are means ± SD. 2-tailed, unpaired Student's t test. ns=not significant.

**F)** Volcano plot of liver proteome of LMKO mice analyzed by mass spectrometry with mitochondrial dynamics proteins in orange (Supplemental Dataset 2).

**G)** Colocalization of DRP1 and TOMM40 in control and LMKO hepatocytes. Mitochondrial labeled in purple with TOMM40 antibody. DRP1 antibody in yellow. Scale bar=50$\mu$m. Mander's overlap coefficient for DRP1. Control n= 28. LMKO n= 23. Data are means ± SD. 2-tailed, unpaired Student's t test, ns=not significant.

**H)** Cellular fractionation and immunoblot analysis of steady levels of DRP1, TOMM40, FIS1, OPA1 and GAPDH proteins in cytosolic and mitochondrial liver fractions from control and LMKO male mice. Immunoblot performed twice with similar results.

**I)** Quantification of proteins in mitochondrial fractions depicted in H). Data are means ± SD. 2-tailed, unpaired Student's t test, ns=not significant.

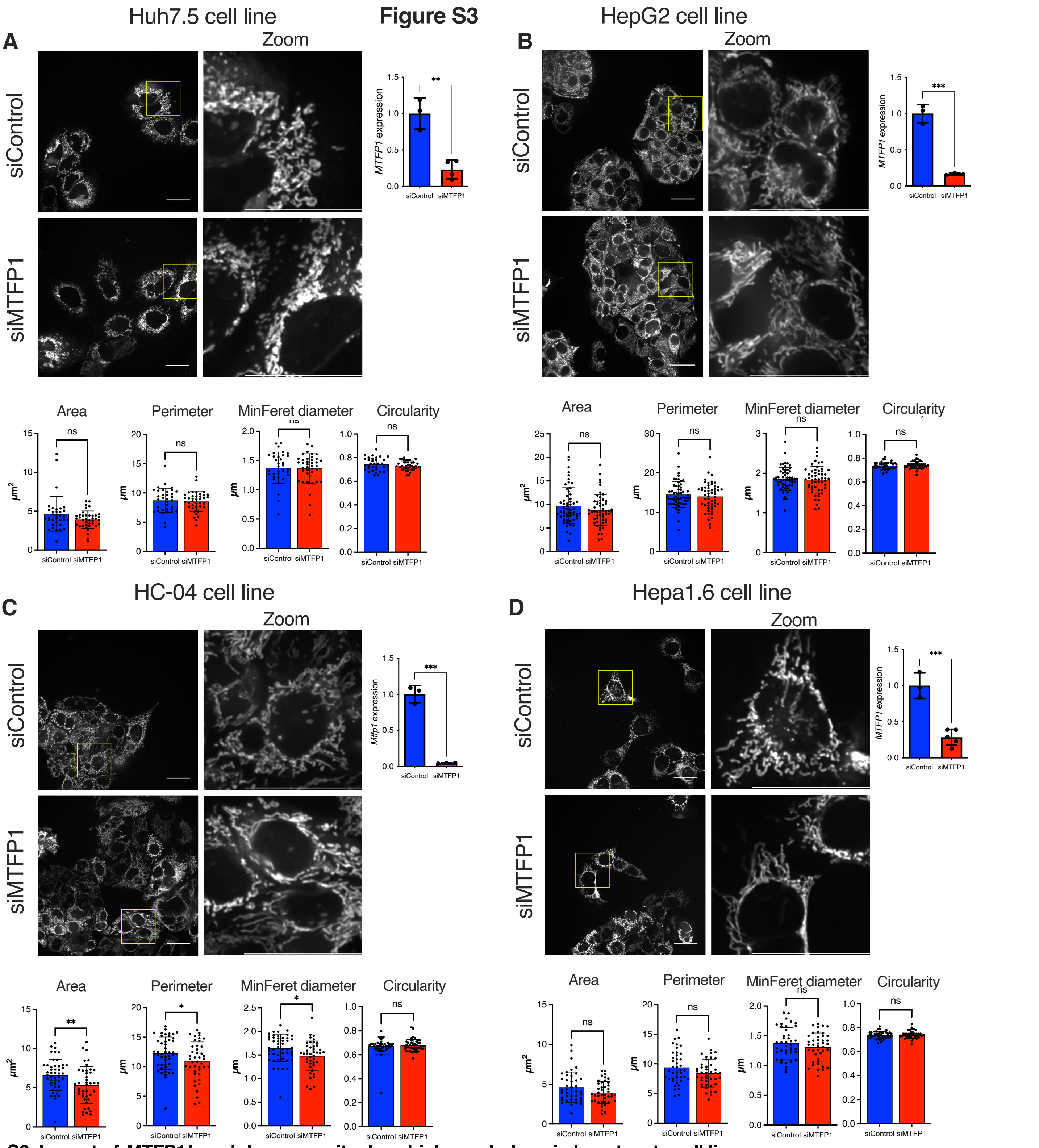


**Figure S3. Impact of *MTFP1* knockdown on mitochondrial morphology in hepatocyte cell lines**

**A)** Representative images of Huh7.5 siControl and siMTFP1 cells. Scale bar=30$\mu$m. Parameters represented area, perimeter, minimum Feret diameter and circularity. Control n= 34. LMKO n= 39. *MTFP1* gene expression in siControl and siMTFP1Huh7.5 cells. β-*ACTIN* gene was used as housekeeping. Control n= 3. LMKO n= 4. Data are means ± SD. 2-tailed unpaired Student's t test. ns=not significant. **: p<0.01.

**B)** Representative images of HepG2 siControl and siMTFP1 cells. Scale bar=30$\mu$m. Parameters represented area, perimeter, minimum Feret diameter and circularity. Control n= 56. LMKO n= 54. *MTFP1* gene expression in siControl and siMTFP1 HepG2 cells. β-*ACTIN* gene was used as housekeeping. Control n= 3. LMKO n= 3. Data are means ± SD. 2-tailed unpaired Student's t test. ns=not significant. ***: p<0.001.

**C)** Representative images of HC-04 siControl and siMTFP1 cells. Scale bar=30$\mu$m. Parameters represented area, perimeter, minimum Feret diameter and circularity. Control n= 47. LMKO n= 42. *MTFP1* gene expression in siControl and siMTFP1 HC-04 cells. β-*ACTIN* gene was used as housekeeping. Control n= 3. LMKO n= 4. Data are means ± SD. 2-tailed unpaired Student's t test. ns=not significant. *: p<0.05. **: p<0.01. ***: p<0.001.

**D)** Representative images of Hepa1.6 siControl and siMTFP1 cells. Scale bar=30$\mu$m. Parameters represented area, perimeter, minimum Feret diameter and circularity. Control n= 41. LMKO n= 40. *Mtfp1* gene expression in siControl and siMTFP1 Hepa1.6 cells. β-*actin* gene was used as housekeeping. Control n= 3. LMKO n= 5. Data are means ± SD. 2-tailed unpaired Student's t test. ns=not significant. ***: p<0.001.

# Figure S4

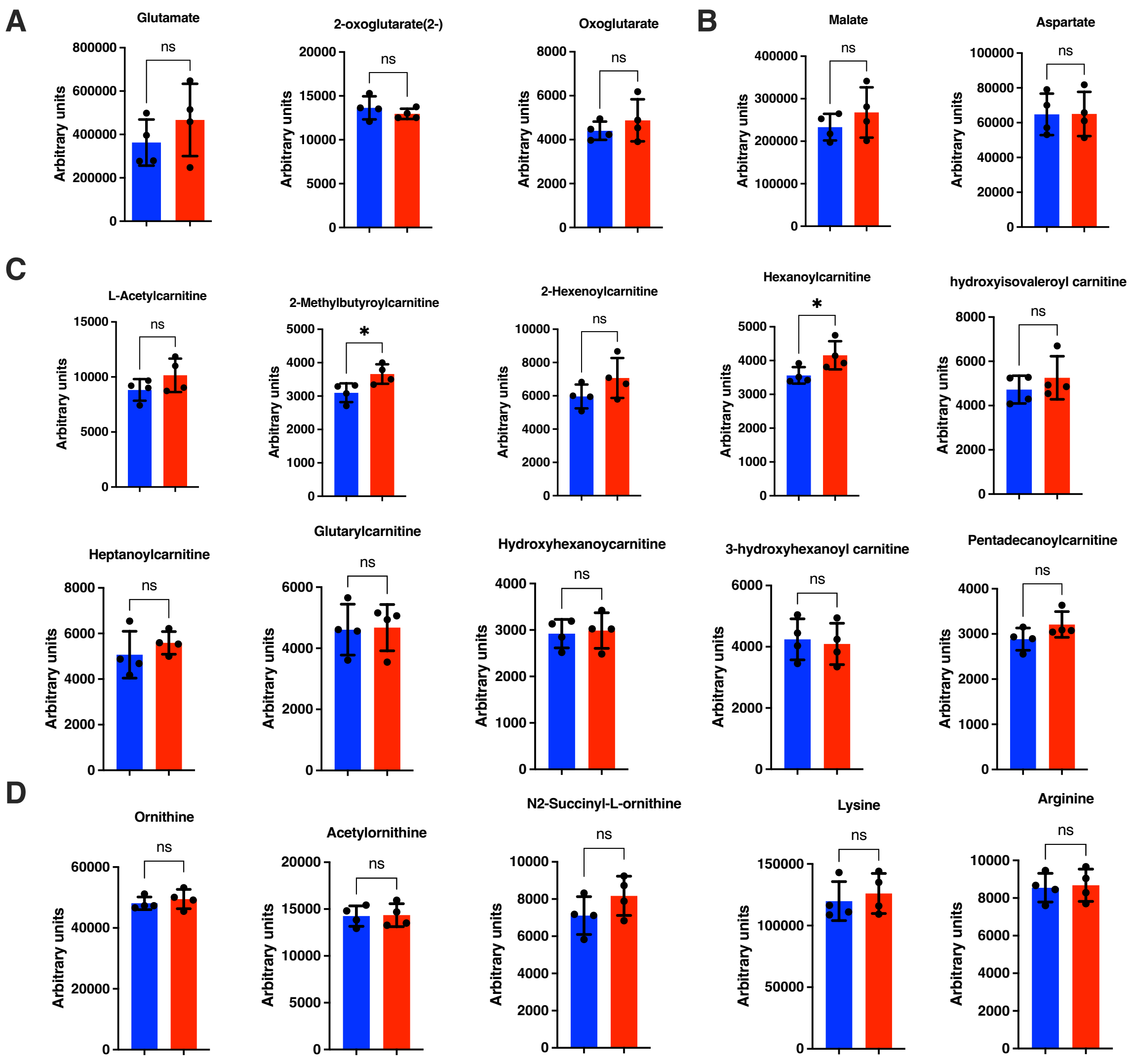


**Figure S4. Metabolomic profiling of livers from Control and LMKO mice fed a NCD**

**A)** Liver metabolites related with SCL25A22 in liver biopsies from Control (n=4) and LMKO (n=4) mice. Data are means ± SD. 2-tailed unpaired Student's t-test, ns=not significant.

**B)** Liver metabolites related with SCL25A11 in liver biopsies from Control (n=4) and LMKO (n=4) mice. Data are means ± SD. 2-tailed unpaired Student's t-test, ns=not significant

**C)** Liver metabolites related with SCL25A20 in liver biopsies from Control (n=4) and LMKO (n=4) mice. Data are means ± SD. 2-tailed unpaired Student's t-test, *: $p<0.05$, ns=not significant

**D)** Liver metabolites related with SCL25A15 in liver biopsies from Control (n=4) and LMKO (n=4) mice. Data are means ± SD. 2-tailed unpaired Student's t-test, ns=not significant.

# Figure S5

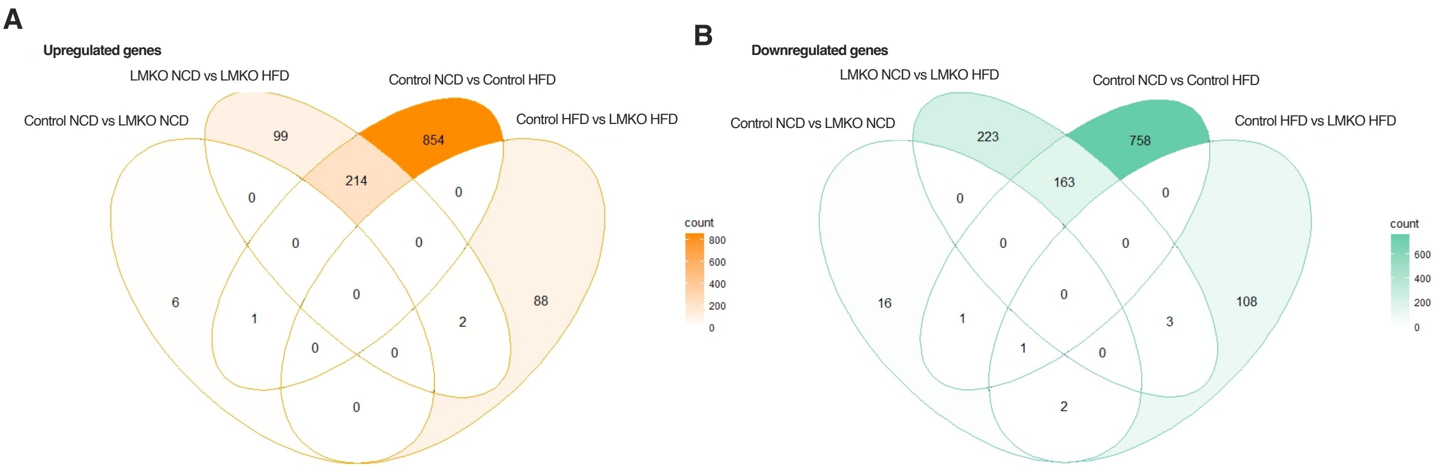


**Figure S5. LMKO mice are protected against diet-induced transcriptomic dysregulation**

**A)** Venn diagram overlap of upregulated genes in LMKO and control mice on normal chow diet (NCD) or high fat diet (HFD) corresponding to Supplemental Dataset 1.

**B)** Venn diagram overlap of downregulated genes in LMKO and control mice on normal chow diet (NCD) or high fat diet (HFD) corresponding to Supplemental Dataset 1.

# Figure S6

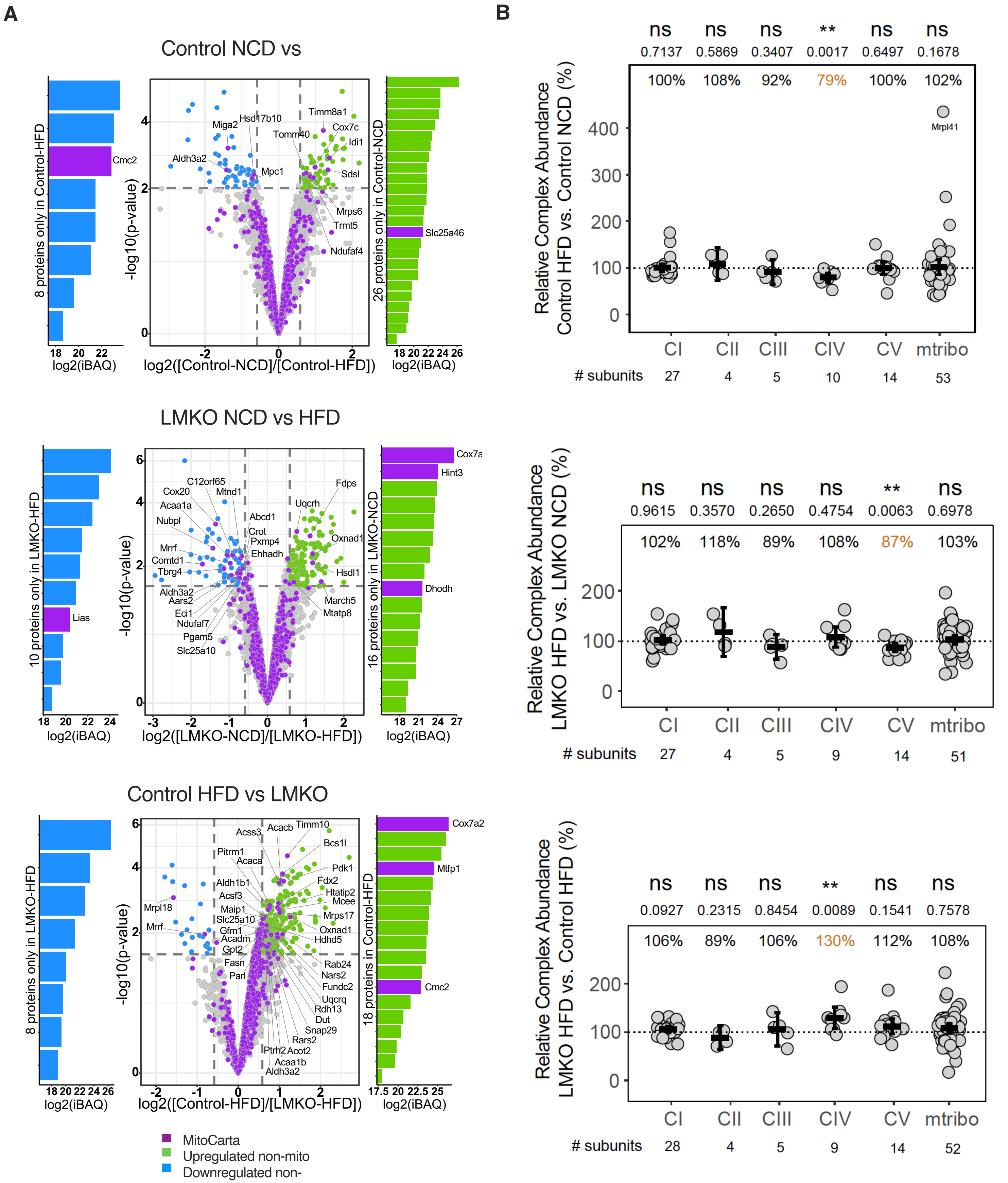


**Figure S6. Diet-induced proteomic remodeling in Control and LMKO mice**

**A)** (Top) Volcano plot of liver proteome of LMKO mice analyzed by mass spectrometry.(Purple). Mitochondrial proteins (MitoCarta 3.0), (Green) Enriched non-mitochondrial proteins more (Blue) Enriched non-mitochondrial proteins corresponding to Supplemental Dataset 3.

**B)** Relative complex abundance (RCA) plot comparing the levels of the OXPHOS complexes (CI-CV) and the mitoribosome (mtribo). The graph represents the relative values of each complex ratio between two groups. The dotted line represents the control mean value of each complex and error bars represent 95% confidence interval of the mean. Paired t-test.** = $p<0.01$, ns= non-significant.

# Figure S7

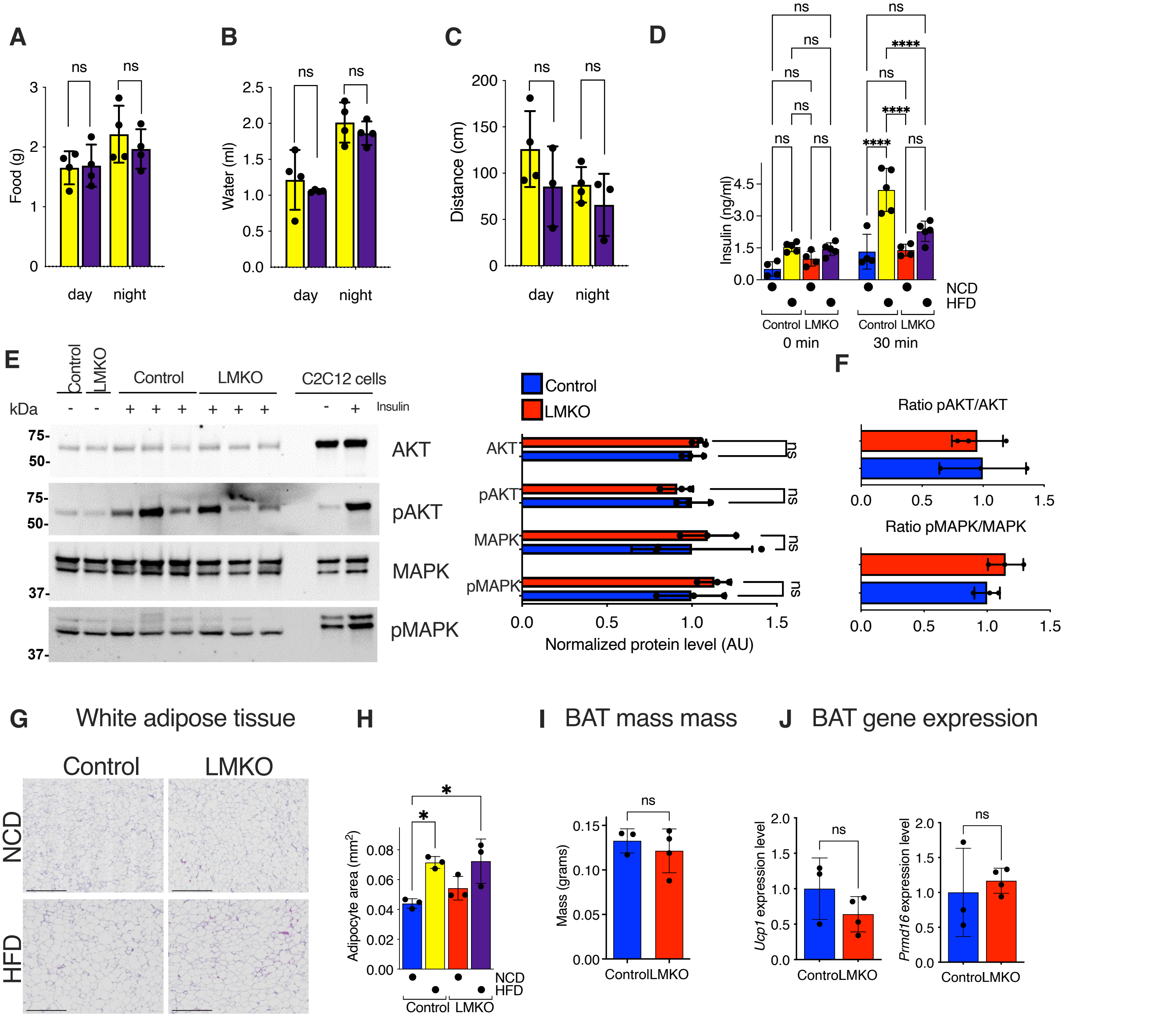


**Figure S7. LMKO mice are protected against diet-induced metabolic dysregulation**

**A)** Mean food consumption per day during dark and light phases in HFD-fed control and LMKO male mice. n=4. Data are means ± SD. 2-tailed unpaired Student's t test, ns=not significant.

**B)** Mean water consumption per day during dark and light phases in HFD-fed control and LMKO male mice n.=4. Data are means ± SD. 2-tailed unpaired Student's t test, ns=not significant.

**C)** Mean distance (3 minutes interval) covered during dark and light phases in HFD-fed control and LMKO male mice. n=4. Data are means ± SD. 2-tailed unpaired Student's t test, ns=not significant

**D)** Plasma insulin levels measured in control and LMKO male mice treated fed with NCD or HFD after 16 hours fasting before (0 min) and after (30 min) glucose injection. n=4-5. Data are means ± SD. 2-way ANOVA, ****: $p<0.0001$

**E)** Immunoblot (left) of AKT and MAPK phosphorylation and total protein levels measured in liver homogenates from fasted control (n=3) and LMKO (n=3) male mice stimulated by insulin for 15 minutes. Non-simulated mice and C2C12 samples were used as a negative and positive controls, respectively. Immunoblot was performed once. Quantification (right) of insulin-stimulated samples. Data are means ± SD, ns=not significant.

**F)** Ratio phosphorylated/total protein levels for AKT and MAPK in liver homogenate from control (n=3) and LMKO (n=3) mice fed a NCD. Data are means ± SD, n2=not significant.

**G)**Representative images of extrahepatic white adipose tissue (eWAT) after H&E staining from control and LMKO male mice fed with NCD or HFD for 16 weeks. n=4-5. Scale bar=100$\mu$m.

**H)** Quantification of adipocyte area in G).Data are means ± SD. 2-way ANOVA, *: $p<0.05$.

**I)** Brown adipose tissue (BAT) mass in control (n=3) and LMKO (n=4) male mice fed a NCD. Data are means ± SD, 2-tailed unpaired Student's t test, ns=not significant.

**J)** Expression levels of *Ucp1* and *Prmd16* in control (n=3) and LMKO (n=4) mice fed a NCD.*Gapdh* gene was used as housekeeping. Data are means ± SD, 2-tailed unpaired Student's t test, ns=not significant.

# Figure S8

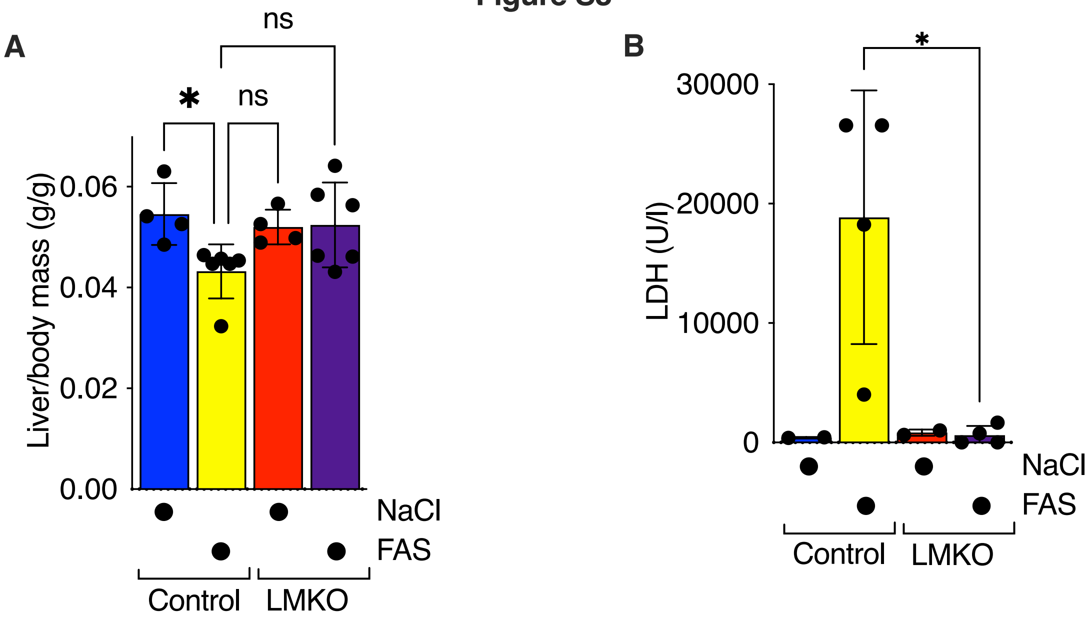


**Figure S8. Hepatic deletion of *Mtfp1* protects mice from FAS-induced liver damage.**

**A)** Lactate dehydrogenase (LDH) plasma levels of 16-week old control and LMKO after treatment with FAS (n=6) or NaCl (n=4) for 24 hours. Data are means ± SD. 2-tailed, unpaired Student's t test. *: p<0.05, ns=not significant.

**B)** Liver to body weight ratio of 16-week old control and LMKO before and after treatment with FAS (n=4) or NaCl (n=2) for 24 hours. Data are means ± SD. 2-way ANOVA, *: p<0.05.

# Figure S9

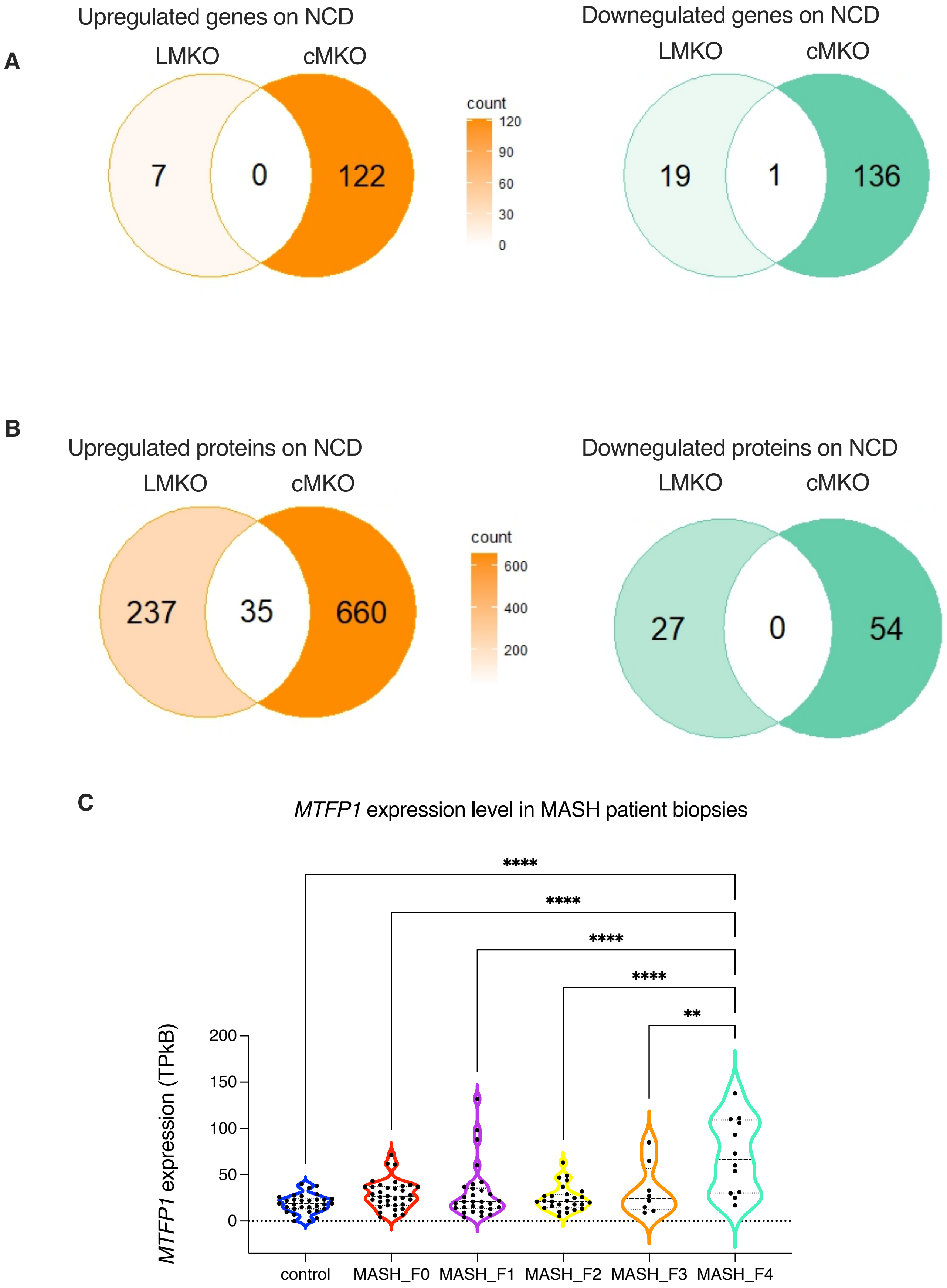


**Figure S9. Differential MTFP1 expression in animal models and human patients**

**A)** Venn diagram comparison of differentially expressed genes in MTFP1-deficient livers and hearts from LMKO and cMKO mice. LMKO data are reported in Supplemental Dataset 1 and cMKO data were reported previously[16].

**B)** Venn diagram comparison of differentially expressed proteins in MTFP1-deficient livers and hearts from LMKO and cMKO mice. LMKO data are reported in Supplemental Dataset 2 and cMKO data were reported previously[16].

**C)** Violin plot of *MTFP1* expression (transcripts per kilobase (TPkB) in MASH human patients biopsies (GSE162694) previously analyzed[103]. F0-F4 scale refers to the severity of fibrosis as previously described[101]. Data are means ± SD. 1-way ANOVA, **: $p<0.01$ ****: $p<0.0001$.
