# Supplementary material for: Mtfp1 ablation enhances mitochondrial respiration and protects against hepatic steatosis
Source: Nat Commun. 2023 Dec 20;14:8474. doi: 10.1038/s41467-023-44143-9 (PMC10733382; doi:10.1038/s41467-023-44143-9)
Supplement: Supplementary file 3 — Description of Additional Supplementary Files [file 41467_2023_44143_MOESM3_ESM.pdf]

Description of Additional Supplementary Files

Supplementary Data 1 - Bulk RNAseq complete datasets for Control and LMKO mice fed a Normal Chow Diet (NCD) or High Fat Diet (HFD) for 16 weeks

Supplementary Data 2 - LFQ proteomics datasets for Control and LMKO mice fed a Normal Chow Diet (NCD) or High Fat Diet (HFD) for 16 weeks

Supplementary Data 3 - MTFP1 interactomics dataset

Supplementary Data 4 - Metabolomics

Supplementary Data 5 - Reagents used in this study including siRNAs, primers, plasmids, mice and antibodies
