# Supplementary material for: Mtfp1 ablation enhances mitochondrial respiration and protects against hepatic steatosis
Source: Nat Commun. 2023 Dec 20;14:8474. doi: 10.1038/s41467-023-44143-9 (PMC10733382; doi:10.1038/s41467-023-44143-9)
Supplement: Supplementary file 9 — Reporting Summary [file 41467_2023_44143_MOESM9_ESM.pdf]

nature portfolio

Corresponding author(s): TIMOTHY WAI
Last updated by author(s): Nov 22, 2023

Reporting Summary

Nature Portfolio wishes to improve the reproducibility of the work that we publish. This form provides structure for consistency and transparency in reporting. For further information on Nature Portfolio policies, see our Editorial Policies and the Editorial Policy Checklist.

Please do not complete any field with "not applicable" or n/a. Refer to the help text for what text to use if an item is not relevant to your study.
For final submission: please carefully check your responses for accuracy; you will not be able to make changes later.

Statistics

For all statistical analyses, confirm that the following items are present in the figure legend, table legend, main text, or Methods section.

n/a	Confirmed	
☐	☒	The exact sample size (n) for each experimental group/condition, given as a discrete number and unit of measurement
☐	☒	A statement on whether measurements were taken from distinct samples or whether the same sample was measured repeatedly
☐	☒	The statistical test(s) used AND whether they are one- or two-sided<br>*Only common tests should be described solely by name; describe more complex techniques in the Methods section.*
☐	☒	A description of all covariates tested
☐	☒	A description of any assumptions or corrections, such as tests of normality and adjustment for multiple comparisons
☐	☒	A full description of the statistical parameters including central tendency (e.g. means) or other basic estimates (e.g. regression coefficient) AND variation (e.g. standard deviation) or associated estimates of uncertainty (e.g. confidence intervals)
☐	☒	For null hypothesis testing, the test statistic (e.g. F, t, r) with confidence intervals, effect sizes, degrees of freedom and P value noted<br>*Give P values as exact values whenever suitable.*
☒	☐	For Bayesian analysis, information on the choice of priors and Markov chain Monte Carlo settings
☒	☐	For hierarchical and complex designs, identification of the appropriate level for tests and full reporting of outcomes
☒	☐	Estimates of effect sizes (e.g. Cohen's d, Pearson's r), indicating how they were calculated

Our web collection on statistics for biologists contains articles on many of the points above.

Software and code

Policy information about availability of computer code

Data collection	Fluorescence microscopy (Harmony 4.9, Perkin Elmer), fluorespirometry (O2k-FlouRespirometer, Oroboros), immunoblotting (Bio-Rad Chemidoc), and electron microscopy (Serial EM), and immunohistochemistry (OlyVIA, Olympus)
Data analysis	Commercially available software was used for the analysis fluorespirometry (Data Lab Oroboros), immunoblotting (Imagelab, Bio-Rad), fluorescence microscopy (Harmony 4.9, Perkin Elmer) (Fiji, ImageJ), and electron microscopy data (3dmod). Statistical analyses were performed using Graph Pad Prism v9.

For manuscripts utilizing custom algorithms or software that are central to the research but not yet described in published literature, software must be made available to editors and reviewers. We strongly encourage code deposition in a community repository (e.g. GitHub). See the Nature Portfolio guidelines for submitting code & software for further information.

Data

Policy information about availability of data

All manuscripts must include a data availability statement. This statement should provide the following information, where applicable:

- Accession codes, unique identifiers, or web links for publicly available datasets
- A description of any restrictions on data availability
- For clinical datasets or third party data, please ensure that the statement adheres to our policy

Source Data are provided with this paper. The datasets generated during the current study are available in the European Nucleotide Archive (NEO) repository and Proteomics Identification Database (PRIDE) (ProteomeXchange Consortium). Accession numbers and the web links are as follows. Bulk RNAseq: (ENA: E-MTAB-12920) (mouse reference genome GRCm39), Liver proteome of mice (PXD041197), and liver interactome data (PXD046262). The total datasets of fluorescence microscopy images generated and analyzed during the current study are not publicly available due to the incompatibility of exporting comprehensible file names linked to cell, treatment, and time identifiers from the Harmony 4.9, Perkin Elmer software but are available from the corresponding author on reasonable request.

Research involving human participants, their data, or biological material

Policy information about studies with human participants or human data. See also policy information about sex, gender (identity/presentation), and sexual orientation and race, ethnicity and racism.

Reporting on sex and gender	N/A
Reporting on race, ethnicity, or other socially relevant groupings	N/A
Population characteristics	N/A
Recruitment	N/A
Ethics oversight	N/A

Note that full information on the approval of the study protocol must also be provided in the manuscript.

Field-specific reporting

Please select the one below that is the best fit for your research. If you are not sure, read the appropriate sections before making your selection.

☒ Life sciences ☐ Behavioural & social sciences ☐ Ecological, evolutionary & environmental sciences

For a reference copy of the document with all sections, see nature.com/documents/nr-reporting-summary-flat.pdf

Life sciences study design

All studies must disclose on these points even when the disclosure is negative.

Sample size	Sample sizes were deemed sufficient, taking into account the literature referencing analogous experiments (Wai et al. Science 2015), as well as the magnitude and consistency of measurable differences between groups, power studies (conducted in partnership with Biostatisticians at the Institut Pasteur), and the available space within animal containment facilities utilized for breeding (BIME) and experimentation (Monod).
Data exclusions	No data points were excluded in this study.
Replication	Animal experiments were independently repeated a minimum of three times using distinct biological samples. Ex vivo and in vitro experiments, encompassing cell death/growth assays, seahorse measurements, and biochemical assays, were conducted on two or three biologically independent samples, consistently yielding reproducible data. It's noteworthy that all replication attempts were successful, and the corresponding replicates have been incorporated into the figure legends for reference.
Randomization	In animal studies, both control and mutant mice were randomly assigned to cages and gender-separated upon weaning. Physiological measurements were taken randomly, cage by cage. For pair-wise experimental measurements conducted in vitro (such as Oroboros, hepatocytes isolation, and electro-physiology), mutant and wild type samples were simultaneously measured. In experiments other than animal studies, samples were allocated randomly between genotypes whenever feasible. However, random allocation wasn't possible when dealing with simultaneous, pairwise measurements (e.g., a machine with only two chambers). In such cases, randomly selected wild type control and mutant samples were simultaneously measured in individual chambers.
Blinding	Blinding was implemented whenever feasible, especially in all animal experiments. However, this practice was not applicable in experiments where the protocols necessitated parallel, pair-wise measurements (as mentioned earlier). In such instances, random wild type and mutant samples were obtained from previously genotyped cells or animals. For cell and tissue image acquisition and analysis, automated processes were employed, ensuring that the experimenter remained blinded to the data. Additionally, all data underwent analysis using unbiased statistical methods.

Reporting for specific materials, systems and methods

We require information from authors about some types of materials, experimental systems and methods used in many studies. Here, indicate whether each material, system or method listed is relevant to your study. If you are not sure if a list item applies to your research, read the appropriate section before selecting a response.

Materials & experimental systems

n/a	Involved in the study
☐	☒ Antibodies
☐	☒ Eukaryotic cell lines
☒	☐ Palaeontology and archaeology
☐	☒ Animals and other organisms
☒	☐ Clinical data
☒	☐ Dual use research of concern
☒	☐ Plants

Methods

n/a	Involved in the study
☒	☐ ChIP-seq
☒	☐ Flow cytometry
☒	☐ MRI-based neuroimaging

Antibodies

Antibodies used

Anti-AKT (#9272,Cell Signalling) (dilution 1:500)
anti-ANT1 (ab110322, Abcam) (dilution 1:500)
anti-ATP5A (ab14748, Abcam) (dilution 1:500)
anti-ATP5B (A21351, ThermoFisher Scientific) (dilution 1:1000)
anti-COX4 (A21348, ThermoFisher Scientific) (dilution 1:500)
anti-cytochrome c (556433,BD Biosciences) (dilution 1:1000)
anti-DRP1 (611112, BD Biosciences) (dilution 1:500)
anti-FIS1 (10956-1-AP, Proteintech Group) (dilution 1:500)
anti-FLAG (F1804, Sigma Aldrich) (dilution 1:1000)
anti-GAPDH (60004-1-Ig, Proteintech Group) (dilution 1:500)
anti-MFN2 (12186-1-AP, Proteintech Group) (dilution 1:1000)
anti-MRPS14 (16301-1-AP, Proteintech Group) (dilution 1:500)
anti-MT-CO2 (55070-1-AP, Proteintech Group) (dilution 1:1000)
anti-MTFP1 (14257-1-AP, Proteintech Group) (dilution 1:1000)
anti-NDUFA9 (ab14713, Abcam) (dilution 1:500)
anti-NDUFB8 (ab110411, Abcam) (dilution 1:1000)
anti-OPA1 (612607, BD Biosciences) (dilution 1:500)
anti-p44/42 MAPK (Erk1/2) (9102S, Cell Signalling) (dilution 1:500)
anti-Phospho-p44/42 MAPK (4370T, Cell Signalling) (dilution 1:500)
anti-Phospho-AKT (#9271, Cell Signalling) (dilution 1:500)
anti-PPIF (18466-1-AP, Proteintech Group) (dilution 1:500)
anti-SDHA (459200, Invitrogen) (dilution 1:500)
anti-SDHB (ab110411, Abcam) (dilution 1:1000)
anti-TIMM22 (14927-1-AP, Proteintech Group) (dilution 1:1000)
anti-TOMM40 (18409-1-AP, Proteintech Group) (dilution 1:500)
anti-UQCRC2 (ab14745, Abcam) (dilution 1:500)
anti-VDAC (PA1-954A, ThermoFisher Scientific) (dilution 1:1000)
anti-VINCULIN (26520-1-AP, Proteintech Group) (dilution 1:500)
anti-Rabbit HRP Conjugated (a120-101p, Bethyl Laboratories) (dilution 1:10000)
anti-Mouse HRP Conjugated (a90-116p, Bethyl Laboratories) (dilution 1:10000)
Anti-Mouse IgG (H+L) Goat, Alexa Fluor 568, (Invitrogen A-11004) (dilution 1:1000)
Alexa Rabblit IgG (H+L) Goat Alexa 488 (Invitrogen A11034) (dilution 1:1000)

Validation

- Anti-AKT (#9272,Cell Signalling) validated for WB by the manufacturer: https://www.cellsignal.com/products/primary-antibodies/akt-antibody/9272?site-search-type=Products&N=4294956287&Ntt=%239272&fromPage=plp&_requestid=3231539
- anti-ANT1 (ab110322, Abcam) validated for WB by the manufacturer: https://www.abcam.com/adenine-nucleotide-translocator-1--2ant-1--2-antibody-5f51bb5ag7-ab110322.html
- anti-ATP5A (ab14748, Abcam) validated for WB by the manufacturer: https://www.abcam.com/adenine-nucleotide-translocator-1--2ant-1--2-antibody-5f51bb5ag7-ab110322.html
- anti-ATP5B (A21351, ThermoFisher Scientific) validated for WB by the manufacturer: https://www.abcam.com/adenine-nucleotide-translocator-1--2ant-1--2-antibody-5f51bb5ag7-ab110322.html
- anti-COX4 (A21348, ThermoFisher Scientific) validated for WB by the manufacturer: https://www.thermofisher.com/antibody/product/OxPhos-Complex-IV-subunit-IV-Antibody-clone-20E8C12-Monoclonal/A21348
- anti-cytochrome c (556433,BD Biosciences) validated for WB by the manufacturer: https://www.bdbiosciences.com/ko-kr/products/reagents/western-blotting-and-molecular-reagents/western-blot-reagents/purified-mouse-anti-cytochrome-c.556433
- anti-DRP1 (611112, BD Biosciences) validated for WB and DRP1 by the manufacturer and in our previous study (PMID: 34014035)
- anti-FIS1 (10956-1-AP, Proteintech Group) validated for WB by the manufacturer and in our previous study (PMID: 34014035) https://www.ptglab.com/products/FIS1-Antibody-10956-1-AP.htm
- anti-FLAG (F1804, Sigma Aldrich) validated for WB by the manufacturer and in our previous study (PMID: 34014035) https://www.sigmaaldrich.com/FR/fr/product/sigma/f1804?gclid=CjwKCAiArOqOBhBmEiwAsgeLmbJ9v1KMtE_wHobmZVCkTAEihCAqo5qbSrsooghThdifewXsApo9shoCaN4QAvD_BwE
- anti-GAPDH (60004-1-Ig, Proteintech Group) validated for WB by the manufacturer: https://www.ptglab.com/products/GAPDH-Antibody-60004-1-Ig.htm
- anti-MFN2 (12186-1-AP, Proteintech Group) validated for WB by the manufacturer: https://www.ptglab.com/products/MFN2-Antibody-12186-1-AP.htm
- anti-MRPS14 (16301-1-AP, Proteintech Group) validated for WB by the manufacturer: https://www.ptglab.com/products/MRPS14-Antibody-16301-1-AP.htm
- anti-MT-CO2 (55070-1-AP, Proteintech Group) validated for WB by the manufacturer: https://www.ptglab.com/products/COX2-Antibody-55070-1-AP.htm
- anti-MTFP1 (14257-1-AP, Proteintech Group) validated for WB by the manufacturer and in our study in knockout mice tissues. https://www.sigmaaldrich.com/FR/fr/product/sigma/sab4301167
- anti-NDUFA9 (ab14713, Abcam) validated for WB by the manufacturer: https://www.abcam.com/ndufa9-antibody-20c11b11b11-ab14713.html
- anti-NDUFB8 (ab110411, Abcam) validated for WB by the manufacturer and in our previous study https://www.abcam.com/products/panels/total-oxphos-human-wb-antibody-cocktail-ab110411.html
- anti-OPA1 (612607, BD Biosciences) validated for WB by the manufacturer and in our previous study (PMID: 34014035) https://www.bdbiosciences.com/en-nz/products/reagents/microscopy-imaging-reagents/immunofluorescence-reagents/purified-mouse-anti-opa1.612607
- anti-p44/42 MAPK (Erk1/2) (9102S, Cell Signalling) validated for WB by the manufacturer: https://www.cellsignal.com/products/primary-antibodies/p44-42-mapk-erk1-2-antibody/9102?site-search-type=Products&N=4294956287&Ntt=9102s&fromPage=plp&_requestid=213206
- anti-Phospho-p44/42 MAPK (4370T, Cell Signalling) validated for WB by the manufacturer: https://www.cellsignal.com/products/primary-antibodies/phospho-p44-42-mapk-erk1-2-thr202-tyr204-d13-14-4e-xp-rabbit-mab/4370?site-search-type=Products&N=4294956287&Ntt=4370t&fromPage=plp&_requestid=212545
- anti-Phospho-AKT (#9271, Cell Signalling) validated for WB by the manufacturer: https://www.cellsignal.com/products/primary-antibodies/phospho-akt-ser473-antibody/9271
- anti-PPIF (18466-1-AP, Proteintech Group) validated for WB by the manufacturer and in our previous study (PMID: 36333300)
- anti-SDHA (459200, Invitrogen) validated for WB by the manufacturer: https://www.thermofisher.com/antibody/product/SDHA-Antibody-clone-2E3GC12FB2AE2-Monoclonal/459200
- anti-SDHB (ab110411, Abcam) validated for WB by the manufacturer and in our previous study https://www.abcam.com/products/panels/total-oxphos-human-wb-antibody-cocktail-ab110411.html
- anti-TIMM22 (14927-1-AP, Proteintech Group) https://www.ptglab.com/products/TIMM22-Antibody-14927-1-AP.htm
- anti-TOMM40 (18409-1-AP, Proteintech Group) validated for WB and ICC by the manufacturer: https://www.ptglab.com/products/TOMM40-Antibody-18409-1-AP.htm
- anti-UQCRC2 (ab14745) validated for WB by the manufacturer and in our previous study (PMID: 34014035)
- anti-VDAC (PA1-954A, ThermoFisher Scientific) validated for WB by the manufacturer:https://www.thermofisher.com/antibody/product/VDAC-Antibody-Polyclonal/PA1-954A%27
- anti-VINCULIN (26520-1-AP, Proteintech Group) validated for WB by the manufacturer: https://www.ptglab.com/products/Vinculin-Antibody-26520-1-AP.htm
- anti-Rabbit HRP Conjugated (a120-101p, Bethyl Laboratories) validated for WB by the manufacturer and in our previous study (PMID: 34014035) https://www.fishersci.com/shop/products/goat-anti-rabbit-igg-heavy/NC0329705
- anti-Mouse HRP Conjugated (a90-116p, Bethyl Laboratories) validated for WB by the manufacturer and in our previous study (PMID:34014035)https://www.fishersci.com/shop/products/goat-anti-mouse-igg-hrp-11/NC9965958?searchHijack=true&searchTerm=a90-116p&searchType=RAPID&matchedCatNo=a90-116p
- Anti-Mouse IgG (H+L) Goat, Alexa Fluor 568, (Invitrogen A-11004) validated for ICC by the manufacturer: https://www.thermofisher.com/antibody/product/Goat-anti-Mouse-IgG-H-L-Cross-Adsorbed-Secondary-Antibody-Polyclonal/A-11004
- Alexa Rabblit IgG (H+L) Goat Alexa 488 (Invitrogen A11034) validated for ICC by the manufacturer: https://www.thermofisher.com/antibody/product/Goat-anti-Rabbit-IgG-H-L-Highly-Cross-Adsorbed-Secondary-Antibody-Polyclonal/A-11034

Eukaryotic cell lines

Policy information about cell lines and Sex and Gender in Research

Cell line source(s)	Primary hepatocytes were isolated in this study from female wild type (Mtfp1+/+) and knockout (Mtfp1-/-) mice. Immortalized human liver cell line: Huh7.5 (a kind gift from Martin Kächele), HepG2(HB-8065) and HC-04 (MRA-975). Immortalized mouse liver cell line: hepa1.6 (CRL-1830). Immortalized mouse muscle cell line: C2C12 (a kind gift from Eric Hajduch).
Authentication	Cells lines were not authenticated beyond the information provided by the supplier.
Mycoplasma contamination	Immortalized cell lines tested negative for mycoplasma.
Commonly misidentified lines (See ICLAC register)	No misidentified cell lines used in this study.

Palaeontology and Archaeology

Specimen provenance	N/A
Specimen deposition	N/A
Dating methods	N/A

☐ Tick this box to confirm that the raw and calibrated dates are available in the paper or in Supplementary Information.

Ethics oversight	N/A

Note that full information on the approval of the study protocol must also be provided in the manuscript.

Animals and other research organisms

Policy information about studies involving animals; ARRIVE guidelines recommended for reporting animal research, and Sex and Gender in Research

Laboratory animals	Mus musculus musculus C57Bl6/NCrl and Albumin-Cre strains were used in this study. The ages ranged from 8 to 24 weeks old and are specifically indicated in the figure legends.
Wild animals	This study did not use wild animals.
Reporting on sex	Male mice were used in the in vivo studies. Both genders were used for the ex vivo experiments: OMICS, Histology, stage bioenergetics, cell death assays, PTP assay.
Field-collected samples	This study did not involve samples collected from the field.
Ethics oversight	All animal experiments were performed according to French legislation in compliance with the European Communities Council Directives (2010/63/UE, French Law 2013-118, February 6, 2013) and those of Institut Pasteur Animal Care Committees (CETEA is Comité d'Ethique en Expérimentation Animale 89).

Note that full information on the approval of the study protocol must also be provided in the manuscript.

Clinical data

Policy information about clinical studies

All manuscripts should comply with the ICMJE guidelines for publication of clinical research and a completed CONSORT checklist must be included with all submissions.

Clinical trial registration	N/A
Study protocol	N/A
Data collection	N/A
Outcomes	N/A

Dual use research of concern

Policy information about dual use research of concern

Hazards

Could the accidental, deliberate or reckless misuse of agents or technologies generated in the work, or the application of information presented in the manuscript, pose a threat to:

No | Yes

☒ ☐ Public health
☒ ☐ National security
☒ ☐ Crops and/or livestock
☒ ☐ Ecosystems
☒ ☐ Any other significant area

Experiments of concern

Does the work involve any of these experiments of concern:

No | Yes

☒ ☐ Demonstrate how to render a vaccine ineffective
☒ ☐ Confer resistance to therapeutically useful antibiotics or antiviral agents
☒ ☐ Enhance the virulence of a pathogen or render a nonpathogen virulent
☒ ☐ Increase transmissibility of a pathogen
☒ ☐ Alter the host range of a pathogen
☒ ☐ Enable evasion of diagnostic/detection modalities
☒ ☐ Enable the weaponization of a biological agent or toxin
☒ ☐ Any other potentially harmful combination of experiments and agents

This checklist template is licensed under a Creative Commons Attribution 4.0 International License, which permits use, sharing, adaptation, distribution and reproduction in any medium or format, as long as you give appropriate credit to the original author(s) and the source, provide a link to the Creative Commons license, and indicate if changes were made. The images or other third party material in this article are included in the article's Creative Commons license, unless indicated otherwise in a credit line to the material. If material is not included in the article's Creative Commons license and your intended use is not permitted by statutory regulation or exceeds the permitted use, you will need to obtain permission directly from the copyright holder. To view a copy of this license, visit http://creativecommons.org/licenses/by/4.0/
